# Computerized Tomography Texture Analysis in the Differential Diagnosis of Intracranial Epidermoid and Arachnoid Cysts

**DOI:** 10.7759/cureus.41945

**Published:** 2023-07-16

**Authors:** Ezel Yaltırık Bilgin, Özkan Ünal, Şahap Törenek, Nazan Çiledağ

**Affiliations:** 1 Radiology, Dr. Abdurrahman Yurtaslan Ankara Oncology Training and Research Hospital, Ankara, TUR

**Keywords:** mri imaging, computer-assisted image processing, ct (computed tomography) imaging, intracranial arachnoid cyst, congenital epidermoid cyst

## Abstract

Purpose: This study evaluated the differences between arachnoid and epidermoid cysts in computerized tomography (CT) texture analysis (TA).

Material and methods: The study included 12 patients with intracranial epidermoid cysts and 26 patients with intracranial arachnoid cysts who were diagnosed with diffusion-weighted magnetic resonance imaging (DW-MRI) and who had undergone an unenhanced CT examination before treatment. The LIFEx application software was used to obtain texture features. Eighty-two texture features from 38 lesions were automatically calculated for each lesion. The Shapiro-Wilk test was used to test the normality of the scores, and the Mann-Whitney U Test was used to test the difference between the groups. Receiver operating characteristic (ROC) curves and multivariate logistic regression modeling examined the parameters' diagnostic performances.

Results: The median age of the patients was 53 years (range: 19-88 years). Eighty-two texture parameters were evaluated in the first order: gray-level co-occurrence matrix (GLCM), gray-level run length matrix (GLRLM), neighbor gray-tone difference matrix (NGTDM), and gray-level size zone matrix (GLSZM) groups. There was a statistically significant difference between the arachnoid cyst and the epidermoid cyst in the variables of compacity, compactness 1, compactness 2, sphericity, asphericity, sum average, coarseness, and low gray-level zone (p<0.05). According to the multiple logistic regression model, it was determined that the sum average in the GLCM group (B=-0.11; p=0.015), coarseness (B= 869.5; p=0.044) in the NGTDM group, and morphological sphericity (B=24.18; p=0.047) were the radiomics variables that increased the probability of epidermoid diagnosis. According to the classification table of the model, the sensitivity rate was found to be 83%, and the specificity rate was found to be 96%. Therefore, the probability of accurate model classification was 92%.

Conclusion: CT TA is a method that can be applied with high diagnostic accuracy in the differential diagnosis of intracranial epidermoid and arachnoid cysts, especially in patients who cannot undergo an MRI examination.

## Introduction

Intracranial arachnoid cysts are benign and mostly asymptomatic lesions that develop in both the intracranial area and the spinal canal [1]. They are usually located in the subarachnoid space and contain cerebrospinal fluid. The incidence of intracranial arachnoid cysts is approximately 1.4% [2,3].

Intracranial epidermoid cysts are congenital lesions that account for approximately 1% of all intracranial lesions [4]. They develop due to the inclusion of ectodermal elements during the closure of the neural tube and typically occur in middle age due to the mass effect on adjacent structures [4].

Arachnoid cysts usually do not require surgery or follow-up. Surgery may be considered if it is thought that it causes symptoms [5,6]. The most appropriate treatment method for epidermoid cysts is complete resection of the lesion and capsule to minimize the risk of malignant transformation [7]. Due to the differences in treatment, a differential diagnosis is essential for these two lesions, which have similar findings on imaging.

As in computed tomography (CT), epidermoid cysts are indistinguishable from arachnoid cysts in most conventional magnetic resonance (MR) sequences. They can be distinguished from arachnoid cysts by showing restricted diffusion in diffusion-weighted imaging (DWI) [3]. However, differential diagnosis is a significant clinical problem for clinicians and radiologists in patients who cannot undergo MRI (for reasons such as cardiac pacemakers, operatively implanted devices incompatible with MRI, metallic foreign bodies, claustrophobia, etc.).

Texture analysis (TA) is part of the radiomics studies. It is a method that quantitatively and objectively evaluates the distribution of gray levels in pixels or voxels in the image space and the heterogeneity of the lesions according to their relationship with each other [8,9]. Our study aimed to evaluate the differences between arachnoid and epidermoid cysts in the CT TA.

## Materials and methods

Study population

The study took place at the radiology department of Dr. Abdurrahman Yurtaslan Ankara Oncology Training and Research Hospital, Ankara, Türkiye. Institutional review board approval was obtained for this retrospective study from Dr. Abdurrahman Yurtaslan Ankara Oncology Training and Research Hospital Ethics Committee (approval number: 2022-12/181), with informed consent being waived. The study included 12 patients with epidermoid cysts and 26 patients with arachnoid cysts who were followed up in our clinic between January 2019 and December 2022, who were diagnosed with diffusion MRI, who had undergone non-contrast CT examination before treatment, and who had a lesion size greater than 1 cm (to prevent errors related to segmentation). Patients without diffusion MR imaging and lesions smaller than 1 cm were excluded from the study.

CT acquisition technique

Brain CT scans of the patients were performed with a 16-slice CT device (GE Revolution, General Electric, Milwaukee, Wisconsin, USA). The examination was performed with routine non-contrast brain CT parameters of 120 kV, 200 mAs, pitch value 0.8, collimation 128 × 0.6 mm, and slice thickness 0.75 mm.

Texture analysis

**Figure 1:**
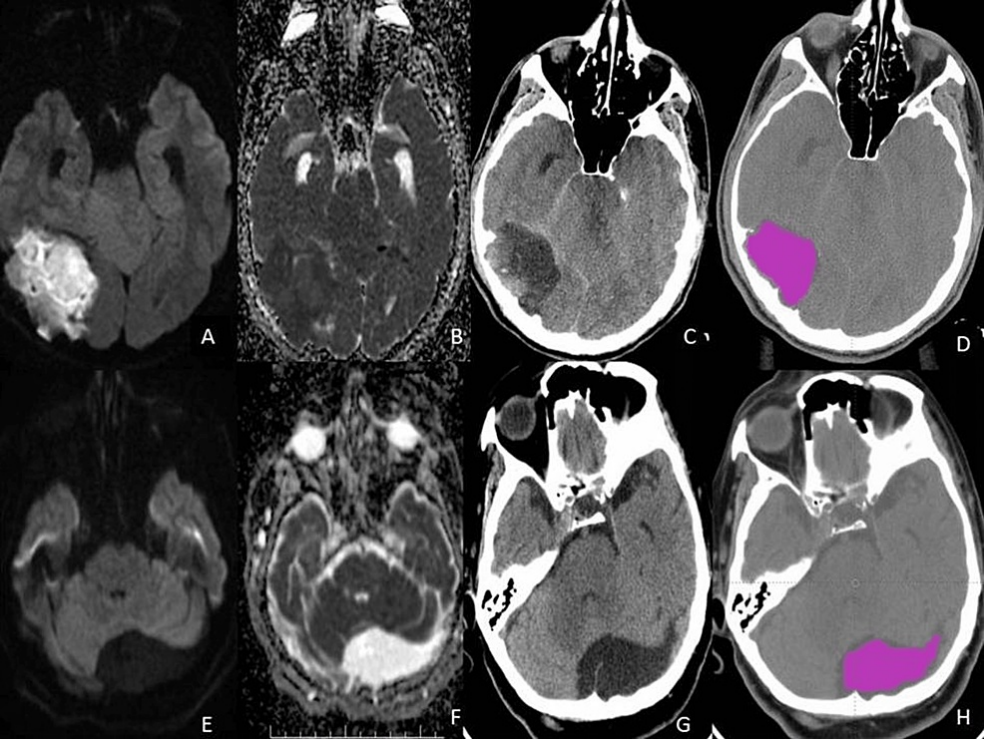
(A): Diffusion-weighted imaging (DWI) of the epidermoid cyst; (B): Apparent diffusion coefficient (ADC) of the epidermoid cyst; (C): Computed tomography (CT) of the epidermoid cyst; (D): Segmentation of the epidermoid cyst; (E): DWI of the arachnoid cyst; (F): ADC of the arachnoid cyst; (G): CT of the arachnoid cyst; (H): Segmentation of the arachnoid cyst

A radiologist with 11 years of neuroimaging experience segmented the lesions. The traditional radiomics approach consists of four steps: image segmentation using regions of interest, feature extraction, feature selection, and prediction. First, the previously selected axial CT slices were retrieved in the Digital Imaging and Communications in Medicine (DICOM) format and imported into texture analysis software (LIFEx application software, version v5.10; www.lifexsoft.org) [6]. Next, 3D volume-of-interest (VOI) data were obtained by drawing 2D regions of interest (ROIs) from each axial CT section containing the lesion. To avoid a partial volume effect in lesions adjacent to vessels and bones, the adjoining 3 mm area was not included in the segmentation. After segmentation, 82 texture features for 38 lesions were automatically calculated for each lesion. Examples of segmented lesions are presented in Figure 1.

During the evaluation of the findings obtained in the study, IBM Statistical Package for Social Sciences (SPSS) version 25.0 (IBM Corp., Armonk, NY, USA) software was used for statistical analysis. Descriptive, graphical, and statistical methods were used to examine whether the scores obtained from each variable were usually distributed. The statistical method used the Shapiro-Wilk test to test the normality of the scores obtained from a continuous variable. In addition to descriptive statistical methods (number, percentage, median, range, etc.), the Mann-Whitney U Test was used to test the difference between the groups while evaluating the study data. The diagnostic performance of the parameters examined in the study in predicting the type of lesion was evaluated by ROC (receiver operating characteristic) curves and multivariate logistic regression modeling. The results of the variables determined by ROC analysis were assessed in the 95% confidence interval, and the significance level was p<0.05.

## Results

A total of 38 patients with cystic brain lesions, consisting of 12 (32%) epidermoid and 26 (68%) arachnoid cysts, were included in the study. The median age of the patients was 53 years (age range: 19-88 years). Eighty-two texture analysis parameters belonging to the first step data, the gray-level co-occurrence matrix (GLCM), gray-level run length matrix (GLRLM), neighbor gray-tone difference matrix (NGTDM), and gray-level size zone matrix (GLSZM) groups, were evaluated statistically.

There was a statistically significant difference between the arachnoid cyst and the epidermoid cyst in the variables of compacity, compactness 1, compactness 2, sphericity, asphericity, sum average, coarseness, and low gray level zone (p<0.05). Accordingly, it was determined that the compacity, asphericity, and sum average levels were lower in epidermoid lesions, and all other variables with significant differences were higher (Table 1).

**Table 1:** Statistically significant computed tomography texture analysis parameters of the lesions GLCM: gray-level co-occurrence matrix; GLRLM: gray-level run length matrix, NGTDM: neighbor gray-tone difference matrix; GLSZM: gray-level size zone matrix

	Epidermoid (n=12; 31.6%)			Arachnoid (n=26; 68.4%)			
	Median	Minimum	Maximum	Median	Minimum	Maximum	p-value
Morphological parameters							
Compacity	20.91	4.17	23.59	25.58	16.71	61.65	<0.001*
Compactness 1	0.03	0.01	0.04	0.02	0.01	0.03	0.014*
Compactness 2	0.25	0.07	0.52	0.17	0.03	0.40	0.014*
Sphericity	0.63	0.50	0.81	0.56	0.31	0.70	0.002*
Asphericity	0.57	0.24	0.70	0.80	0.35	2.23	<0.001*
GLCM							
Sum average	31.57	0.46	68.29	56.18	11.17	86.61	0.033*
NGTDM							
Coarseness	0.00282	0.00027	0.01408	0.00068	0.00003	0.00355	0.004*
GLSZM							
Low gray-level zone	0.0049	0.0017	0.068	0.0032	0.0011	0.059	0.041*

**Figure 2:**
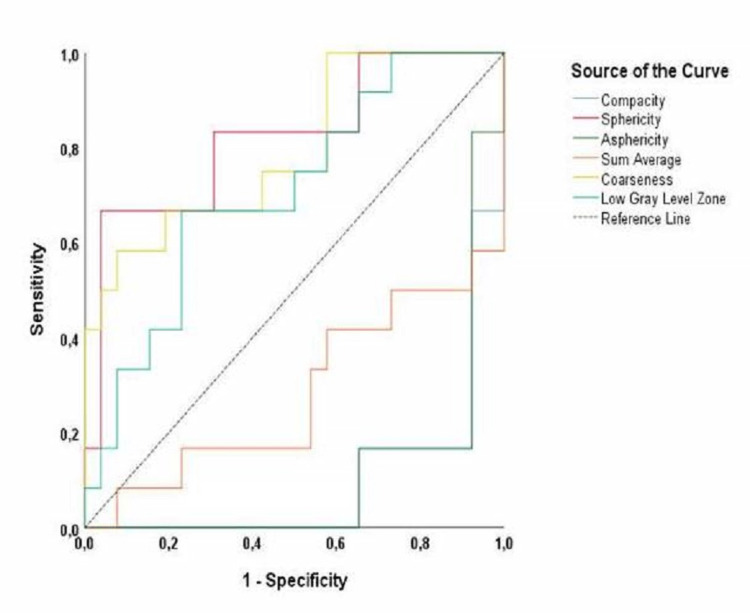
ROC curves of CT texture analysis parameters ROC: receiver operating characteristic curve; CT: computed tomography

As a result of ROC analysis, positive cut-off, sensitivity, and specificity analysis results of radiomics variables of the lesion area with an area under the ROC curve (AUC) value above 0.7 determined in the differential diagnosis of the arachnoid cyst and epidermoid cyst are presented (Table 2, Figure 2).

**Table 2:** The ROC analysis of CT texture analysis parameters in the differential diagnosis of the lesion NGTDM: neighbor gray-tone difference matrix; GLSZM: gray-level size zone matrix; GLCM: gray-level co-occurrence matrix; ROC: receiver operating characteristic curve; CT: computed tomography; CI: confidence interval

Variables	AUC	95% CI	p-value	Cut-off	Sensitivity	Specificity
Sphericity	0.821	(0.667-0.974)	0.002	0.616	0.67	0.96
NGTDM-coarseness	0.795	(0.636-0.954)	0.004	0.0025	0.58	0.92
GLSZM-low gray-level zone	0.708	(0.533-0.884)	0.041	0.0046	0.67	0.77
Compacity	0.904	(0.805-1.000)	<0.001	21.98	0.92	0.83
Asphericity	0.891	(0.784-0.998)	<0.001	0.62	0.92	0.83
GLCM sum average	0.718	(0.525-0.911)	0.033	22.77	0.92	0.50

The ROC analysis was performed by applying the multivariate logistic regression analysis established with the Enter method that included the CT texture analysis variables, which had a statistically significant relationship in the differential diagnosis of the lesion type, had high AUC values, and provided multiple regression assumptions. The regression analysis results showed that the model determination coefficient was R2 (Nagelkerke) = 0.75. Since the p-value of the model (F= 28.868, p<0.001) was smaller than the ɑ value, the model was found to be significant at the 95% confidence level. Furthermore, according to the multiple logistic regression model, it was determined that the sum average in the GLCM group (B=-0.11; p=0.015), coarseness (B= 869.5; p=0.044) in the NGTDM group, and morphological sphericity (B=24.18; p=0.047) were the radiomics variables that increased the probability of epidermoid diagnosis (Table 3).

**Table 3:** CT texture analysis parameters in the differential diagnosis of the lesion (the results of the multivariate logistic regression model analysis) CT: computed tomography; B: unstandardized beta; SE: standard error

Variables	B	SE	Wald	p-value
Sum average	-0.113	0.046	5.902	0.015*
Coarseness	869.514	430.86	4.073	0.044*
Sphericity	24.179	12.164	3.951	0.047*
Constant	12.316	6.262	3.869	0.049

According to the classification table of the model, the sensitivity rate was found to be 83%, and the specificity rate was found to be 96%. Therefore, the probability of accurate model classification was 92%. Furthermore, the model accurately predicted epidermoid cysts' presence by 92% with the sum average, coarseness, and sphericity variables (Table 4, Table 5).

**Table 4:** Classification table of the logistic model created with CT texture analysis parameters in the differential diagnosis of the lesion

Radiological	Epidermoid	Arachnoid	Total
Epidermoid	10	1	11
Arachnoid	2	25	27
Total	12	26	38

**Table 5:** Diagnostic evaluation of the CT texture analysis PPV: positive predictive value; NPV: negative predictive value; CT: computed tomography

Diagnostic tests	
Sensitivity	83.3%
Specificity	96.2%
PPV	90.9%
NPV	92.6%
Accuracy	92.1%

## Discussion

Texture analysis is part of radiomics studies. It is a method that quantitatively and objectively evaluates the distribution of gray levels in pixels and voxels in images within the image space and the heterogeneity of the lesions according to their relationship with each other [8,10,11].

The differential diagnosis of these lesions is clinically significant because intracranial epidermoid and arachnoid cysts have different treatment methods [7].

Epidermoid cysts are distinguished from arachnoid cysts by showing restricted diffusion in the diffusion MRI [12,13]. However, differential diagnosis is an important clinical problem in patients who cannot undergo an MRI examination (patients with cardiac implantable electronic devices, metallic intraocular foreign bodies, implantable neurostimulation systems, cochlear implants, catheters with metallic touches, magnetic dental implants, claustrophobia, etc.) [14].

The results of our study, in which we aimed to make the differential diagnosis of intracranial epidermoid and arachnoid cysts with TA on the basis of fast and easily obtainable CT images, are remarkable.

The current study shows a significant difference between the two types of cysts in eight parameters. Sphericity and asphericity are morphologic features to measure the tumor's shape [15]. Sphericity may be used to differentiate tumor phenotypes independent of tumor size among all morphologic features. Studies in the literature show that it is a prognostic parameter in oral and lung cancers [16]. Like sphericity, compactness 1 and compactness 2 measure how compact the tumor’s shape is relative to a sphere (closest). Compacity measures how compact the volume of interest is. In the literature, some studies show that compacity is one of the parameters that can predict grade in gastrointestinal stromal tumor cases [17].

The GLCM sum average measures the mean of the gray-level sum distribution of the image. The NGTDM coarseness is a parameter that can be interpreted as a measure of the spatial rate of change between the intensity levels of adjacent voxels [18]. The GLSZM low gray level zone quantifies gray level zones in an image. The gray level zone is classified as the number of connected voxels that share the same gray level intensity [15].

As a result of multiple logistic regression analyses, modeling can distinguish between epidermoid and arachnoid cysts with 96.2% specificity and 92.1% accuracy.

The results of our study will enable the differential diagnosis and determination of the treatment options for epidermoid cysts with CT images in patients who cannot undergo an MRI examination. In addition, it allows differential diagnosis from CT images obtained from incidental cysts detected in patients undergoing CT examination for other reasons. Thus, the need for MRI and associated costs can be reduced.

There is no other study in English literature evaluating the value of CT texture analysis in the differential diagnosis of epidermoid and arachnoid cysts. In addition, the increase in radiomics applications and their clinical use in recent years further increases the value of our study.

The limitations of our study were the retrospective study design and the small number of patients. However, supporting our findings with multicenter studies, including a larger number of patients, will also pave the way for routine clinical applications.

## Conclusions

Computerized tomography (CT) texture analysis (TA) is a highly accurate method for the differential diagnosis of intracranial epidermoid and arachnoid cysts. The utilization of CT TA and the integration of radiomics studies hold great promise in enhancing the diagnostic process for these cystic lesions. With further support from multicenter studies, CT TA has the potential to become a valuable tool in routine clinical applications for the differential diagnosis of intracranial cysts.

The findings of this study highlight the significant advancements that CT and TA bring to the field of neuroimaging. By extracting and analyzing texture features from CT images, clinicians can gain valuable insights into the underlying histological composition and structural differences between intracranial epidermoid and arachnoid cysts. The addition of radiomics algorithms further enhances diagnostic accuracy and provides standardized assessments. As more research is conducted and the findings are validated, CT TA has the potential to revolutionize the differential diagnosis of intracranial cysts, leading to improved patient care and treatment outcomes.
